# Update: Dura Mater Graft–Associated Creutzfeldt-Jakob Disease — Japan, 1975–2017

**DOI:** 10.15585/mmwr.mm6709a3

**Published:** 2018-03-09

**Authors:** Ryusuke Ae, Tsuyoshi Hamaguchi, Yosikazu Nakamura, Masahito Yamada, Tadashi Tsukamoto, Hidehiro Mizusawa, Ermias D. Belay, Lawrence B. Schonberger

**Affiliations:** ^1^Division of Public Health, Center for Community Medicine, Jichi Medical University, Shimotsuke, Japan; ^2^Department of Neurology and Neurobiology of Aging, Kanazawa University Graduate School of Medical Sciences, Kanazawa, Japan; ^3^Department of Neurology, National Center Hospital, National Center of Neurology and Psychiatry, Tokyo, Japan; ^4^Prion and Public Health Office, Division of High-Consequence Pathogens and Pathology, National Center for Emerging and Zoonotic Infectious Diseases, CDC.

Creutzfeldt-Jakob disease (CJD) is a fatal neurodegenerative disorder that, according to the most well accepted hypothesis (*1*), is caused by replicating, transmissible, abnormal forms of a host-encoded prion protein (prions). Most CJD cases occur spontaneously (sporadic CJD) or are inherited (genetic CJD). Iatrogenic CJD can occur after exposure to prion-contaminated instruments or products in medical/surgical settings. Cadaveric dura mater graft–associated CJD (dCJD) accounts for a common form of iatrogenic CJD. This report summarizes the epidemiologic features of 154 cases of dCJD identified in Japan during 1975–2017; these cases account for >60% of dCJD cases reported worldwide (*1*,*2*). The unusually high prevalence of dCJD in Japan was first reported in 1997 (*3*). In 2008, a single brand of graft (Lyodura [B. Braun Melsungen AG, Melsungen, Germany]), frequently used as a patch in neurosurgical procedures, was identified as the probable vehicle of transmission (*4*). No international recall of the implicated Lyodura occurred, the product had a relatively long shelf life, and the grafts were used frequently in Japanese patients with non–life-threatening conditions (*4*,*5*). Since 2008, additional cases have been ascertained, reflecting the identification of previously missed cases and the occurrence of new cases with longer latency periods (interval from exposure to symptom onset) for dCJD (up to 30 years), underscoring the importance of maintaining surveillance for dCJD.

In 1996, after the first report of variant CJD (the human prion disease caused by the agent of bovine spongiform encepathalopathy [“mad cow disease”]) in the United Kingdom (*6*), the nongovernmental Japanese CJD Surveillance Committee (J-CJDSC), with support from the Japanese Ministry of Health, Labour, and Welfare, conducted a preliminary nationwide mail survey to identify cases of human prion disease in Japan; since 1999, J-CJDSC has maintained a national CJD registry (*7*). J-CJDSC members investigate each reported suspected CJD case in cooperation with CJD specialists in each prefecture. The methods for identifying dCJD cases in Japan have been described previously (*5*,*7*,*8*). All identified CJD cases, including cases of dCJD, are entered into the J-CJDSC database, which contains demographic and clinical information, including a detailed history of any surgical procedures and international travel and CJD laboratory test results (including cerebrospinal fluid analyses and genetic testing) (*7*).

Among 829 identified physician-diagnosed cases of CJD during 1979–May 1996, a total of 43 (5%) patients had received a dura mater graft as part of a surgical procedure (typically a patch during neurosurgery); 41 (95%) of these dCJD patients had received a Lyodura graft (*3*). A 1987 U.S. investigation of a dCJD case found that Lyodura produced before May 1987 carried an unusually high risk for dCJD because of the contamination-prone method of production (*9*,*10*); after that report, the manufacturer reported revising its collection and processing procedures to reduce the CJD transmission risk.

By 2008, a total of 132 dCJD cases had been reported in Japan, and among 120 (91%), Lyodura was identified as the probable vehicle of transmission; the graft brand for the other 12 dCJD patients was unknown (*4*). By the end of 2017, the J-CJDSC database included 154 patients with dCJD, including an additional 22 patients identified since the last report (*4*).

Among 154 dCJD patients, receipt of a Lyodura graft was documented in 140 (91%); the brand of dural graft received by 14 patients was not identified. The most common medical conditions for which patients received the cadaveric dura mater grafts were brain tumors (including meningioma) (69; 45%), facial palsy or trigeminal neuralgia (26; 17%), and brain hemorrhage (25; 16%). Less common conditions included intracranial aneurysm (10; 6%), unspecified anomalies (eight; 5%), intracranial hematoma (seven; 5%), trauma (seven; 5%), and other (two; 1%). The median age at symptom onset among dCJD patients was 58 years (range = 15–81 years; mean = 56 years); 89 (58%) patients were female. All patients had received their dura mater graft during 1975–1993 (Figure 1) (Figure 2), and dates of illness onset ranged from 1985 to 2016.

**Figure 1:**
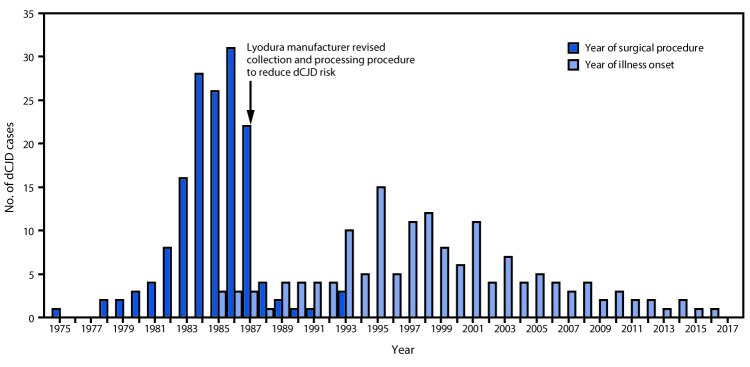
Number of cases (N = 154) of dura mater graft–associated Creutzfeldt-Jakob disease (dCJD), by year of neurosurgical procedure and year of symptom onset — Japan, 1975–2017

**Figure 2:**
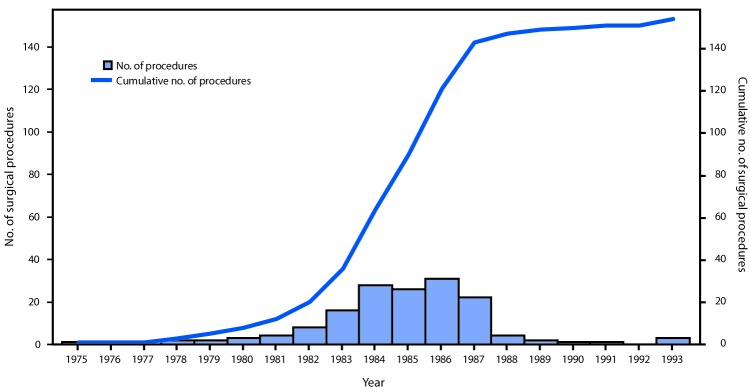
Number of surgical procedures linked to cases of dura mater graft-associated Creutzfeldt-Jakob disease (dCJD),* by year of surgical procedure — Japan, 1975–1993^†^ * Among 154 dura mater graft procedures, the brand was documented as Lyodura in 140 (91%). ^†^ The manufacturer of Lyodura reported that it revised its collection and processing procedures in May 1987 to reduce the risk for CJD contamination; the recommended shelf life for Lyodura was 5 years.

Although the shelf life of Lyodura established by the manufacturer was 5 years, three dCJD patients had surgical procedures in 1993, at least 6 years after the company had changed their collection and processing procedures. J-CJDSC determined that all three patients had received a Lyodura graft, and that at least one of the grafts was processed before 1987, and had therefore expired (the processing date of the second and third patients’ grafts are unknown). Eleven (7%) dCJD patients identified by J-CJDSC received grafts during 1988–1993 (Figure 2), including eight during 1988–1991, indicating that they might have received unexpired Lyodura produced before the company changed its processing procedures in 1987. In 1997, a case occurred in a patient with a history of two neurosurgical procedures in 1991. Investigation by J-CJDSC revealed that the patient had received a graft produced before 1987 during the first procedure. None of the dCJD cases identified to date received a dural graft after 1993.

In Japan, it is estimated that 20,000 persons received a Lyodura graft each year during 1983–1987, approximately 50 times more than the estimated number of U.S. recipients (*4*). During this period, 123 Japanese patients who subsequently developed dCJD had surgical procedures, including 114 (93%) who had documentation of receipt of a Lyodura graft (the graft brand of the other nine patients was unknown), indicating that the risk for developing dCJD within 30 years of receiving a Lyodura graft in Japan was at least one per 877 (i.e., 114 dCJD cases per 100,000 Lyodura graft recipients). In this analysis, both the median and mean intervals from receipt of dural graft to illness onset (latency period) were 13 years (range = 1–30 years) (Figure 3). Since the update in 2008, 11 of the 22 newly reported dCJD cases have had latency periods exceeding 24 years, the longest interval reported in 2008 (*4*) (Figure 3). In three of these 11 cases, the latency period was 30 years, the longest reported to date.

**Figure 3:**
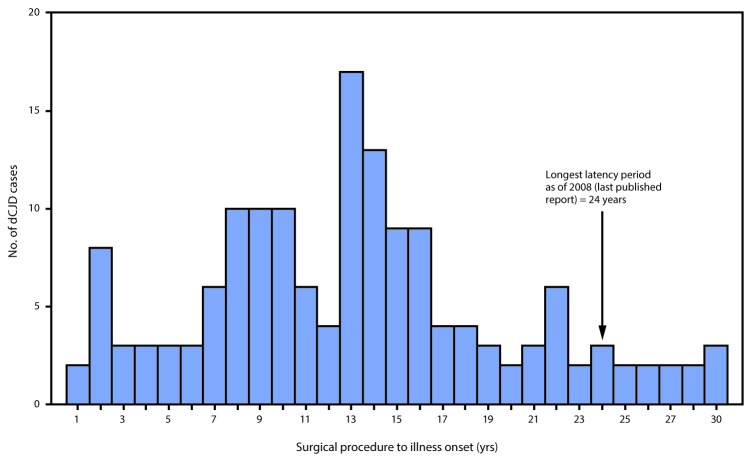
Interval from surgical procedure to illness onset* in 154 cases of dura mater graft–associated Creutzfeldt-Jakob disease — Japan, 1975–2017 * Median = 13 years; range = 1–30 years.

## Discussion

A comprehensive 2012 global summary of dCJD cases by country (*2*) reported that 142 (62%) of 228 cases of dCJD described worldwide occurred in Japan, and that at least one dCJD case was reported from 20 other countries. In the United States, four cases attributed to dura mater grafts have been identified; three were linked to a Lyodura graft produced before 1987, and one to a different commercially produced cadaveric dura mater graft. Lyodura grafts produced before 1987 were widely distributed to many countries, but most frequently to Japan. 

During the U.S. investigation of the first Lyodura-associated CJD case in 1987 (*9*,*10*), investigators learned that the company mixed dura from multiple donors during batch processing of single lots and sterilized the grafts with gamma irradiation, a procedure that does not inactivate prions (*10*). A Lyodura representative also reported that the company did not maintain records identifying donors, so they could not be traced. Lyodura was only available to U.S. hospitals by mail if ordered from a non-U.S. distributor because the manufacturer did not produce the product for distribution in the United States.

Owing to Lyodura’s 5-year shelf life, it is likely that the eight dCJD patients in Japan who received Lyodura during 1988–1991 received grafts produced before the company changed its processing procedures in 1987. In addition, the three patients who received a graft in 1993 all received Lyodura grafts, one of which was documented to be an expired graft processed before 1987.

Age at onset of dCJD depends on the patient’s age at receipt of a dural graft and the latency period. Although the latency period varies among patients, currently available data indicate that the upper limit is at least 30 years, which is longer than has been reported previously (*4*). The most recently diagnosed case, for example, occurred in a patient who received Lyodura during surgery for a craniopharyngioma in 1985 at age 27 years and developed dCJD 30 years later in 2015.

The findings in this report are subject to at least four limitations related to ascertainment of dCJD cases. First, because it is possible that dCJD patients with an unknown brand of dural graft did, in fact, receive Lyodura, it is likely that one dCJD case per 877 Lyodura recipients is an underestimate of the proportion of dCJD patients with Lyodura-related CJD. Second, the risk for a Lyodura-related CJD infection among dural graft recipients is unknown because many infected patients likely died from other causes before developing CJD. Third, additional dCJD cases related to receipt of Lyodura might still occur. The increased use of Lyodura in Japan is the most likely reason for the unusually high number of dCJD cases in Japan (*4*), although only estimates of the numbers of recipients in Japan and other countries, including the United States, are available. Finally, the medical conditions for which dura mater grafts were used in Japan differed from those in other countries (*5*): patients with dCJD in Japan more frequently received dura mater grafts for non–life-threatening conditions than did patients in other countries (*5*).

The cases described in this report indicate that recipients of prion-contaminated grafts could remain at risk for CJD for at least 30 years after receiving grafts. Given the known potential for even longer latency periods for prion diseases, this outbreak is expected to continue. The dCJD cases underscore the importance of establishing measures to eliminate or greatly reduce the possibility of CJD transmissions (e.g., strict donor screening, appropriate record keeping, prevention of cross-contaminations, and ideally, the use of validated sterilization methods) whenever human tissues, particularly of cadaveric origin, might be used to treat other patients. In addition, a system of human disease surveillance to detect the possible emergence of new sources of prion disease transmissions is needed. Furthermore, physicians maintaining a high index of suspicion for unusual prion disease cases, as well as a system of human disease surveillance to detect the emergence of new sources of prion disease transmissions, is needed to enable the prevention of infections Finally, maintaining surveillance for CJD in Japan is important to better assess the impact of the outbreak of dCJD and to identify additional cases.

**Summary**

**What is already known about this topic?**

During 1975–2008, a total of 132 cases of dura mater graft-associated Creutzfeldt-Jakob disease (dCJD), a fatal neurodegenerative disease caused by replicating, transmissible prion proteins, had been identified in Japan and accounted for >60% of patients worldwide with dCJD. This relatively high number of cases was most likely related to the increased use in Japan of the primary vehicle of transmission, Lyodura brand cadaveric grafts produced before May 1987, when the manufacturer changed its production process to reduce the risk for prion transmission.

**What is added by this report?**

During 2008–2017, an additional 22 dCJD patients, with onset from 1985 through 2016, were identified in Japan, resulting in 154 dCJD patients in Japan. No new dCJD patient whose surgery occurred after 1993 has been identified. However, the latency period is now known to be at least 30 years and because of the known potential for even longer latency periods for prion diseases, this outbreak is likely to continue.

**What are the implications for public health practice?**

The dCJD outbreak underscores the importance of strict screening of donors, appropriate record keeping, avoidance of comingling of grafts, and ideally, the use of validated sterilization procedures whenever dura mater grafts are manufactured. The long latency (decades) of human prion diseases can pose challenges to the detection of new sources of infection and highlights the need to recognize prion disease outbreaks and implement preventive measures as early as possible.
