# *Erratum: *Vol. 67, No. 9

**DOI:** 10.15585/mmwr.mm6712a7

**Published:** 2018-03-30

In the report “Update: Dura Mater Graft–Associated Creutzfeldt-Jakob Disease — Japan, 1975–2017,” on page 276, an error occurred in Figure 3. The corrected figure is as follows:

**Figure 3:**
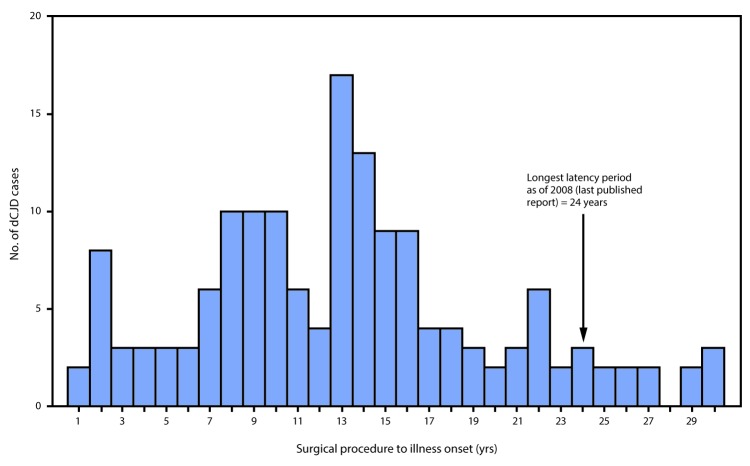
Interval from surgical procedure to illness onset* in 154 cases of dura mater graft–associated Creutzfeldt-Jakob disease — Japan, 1975–2017 * Median = 13 years; range = 1–30 years.

